# BRITER: A BMP Responsive Osteoblast Reporter Cell Line

**DOI:** 10.1371/journal.pone.0037134

**Published:** 2012-05-14

**Authors:** Prem Swaroop Yadav, Paritosh Prashar, Amitabha Bandyopadhyay

**Affiliations:** Department of Biological Sciences and Bioengineering, Indian Institute of Technology, Kanpur, India; Baylor College of Medicine, United States of America

## Abstract

**Background:**

BMP signaling pathway is critical for vertebrate development and tissue homeostasis. High-throughput molecular genetic screening may reveal novel players regulating BMP signaling response while chemical genetic screening of BMP signaling modifiers may have clinical significance. It is therefore important to generate a cell-based tool to execute such screens.

**Methodology/Principal Findings:**

We have established a BMP responsive reporter cell line by stably integrating a BMP responsive dual luciferase reporter construct in the immortalized calvarial osteoblast cells isolated from tamoxifen inducible *Bmp2*; *Bmp4* double conditional knockout mouse strain. This cell line, named BRITER (BMP Responsive Immortalized Reporter cell line), responds robustly, promptly and specifically to exogenously added BMP2 protein. The sensitivity to added BMP may be further increased by depleting the endogenous BMP2 and BMP4 proteins.

**Conclusion:**

As the dynamic range of the assay (for BMP responsiveness) is very high for BRITER and as it responds specifically and promptly to exogenously added BMP2 protein, BRITER may be used effectively for chemical or molecular genetic screening for BMP signaling modifiers. Identification of novel molecular players capable of influencing BMP signaling pathway may have clinical significance.

## Introduction

Bone Morphogenetic Protein (BMP) activity is the molecular basis of the intrinsic regenerative ability of bone [1]. BMPs are members of the transforming Growth Factor-β (TGF-β) superfamily of signaling molecules [2]. So far 20 different BMP molecules have been identified in the humans [3], [4] of which BMP2, BMP4, BMP5, BMP6 and BMP7 are characterized as osteogenic BMPs due to their ability to induce ectopic bone formation [5]. It is known that Bone Morphogenetic Proteins (BMPs) are necessary and sufficient for bone formation [1], [6]. BMP ligands bind to typeI and typeII serine/threonine kinase cell surface receptors. Upon ligand binding, the typeII receptors phosphorylate type I receptors which in turn phosphorylate a group of transcription factors known as receptor-regulated SMADs (R-SMADs) i.e. SMAD1, SMAD5 or SMAD8. Phosphorylated R-SMADs form a heterodimer with SMAD4 and translocate into the nucleus and regulate transcription of a host of BMP downstream target genes [7], [8], [9]. One well characterized BMP downstream target is inhibitor of differentiation-1 (Id1). Korchynski et. al. characterized the promoter region of Id1 and defined a minimal DNA sequence that is responsive to BMP signaling and named it as BMP responsive element (BRE) [10]. BRE consists of SMAD binding elements (SBEs) along with a GGCGCC palindrome. Multimerization of these elements generates a sensitive, BMP-specific enhancer [10]. BRE has been well characterized in different laboratories and in different cell lines [11], [12], [13], [14]. It has been demonstrated that BRE responds specifically to BMP signaling and not to the related pathways such as TGF-β signaling [10].

Occurrence of bone fracture and spinal injuries is quite commonplace. While most fractures heal on their own due to the intrinsic regenerative capacity of bone, a significant problem arises for complex fractures particular those in the elderly. Standard of care for complex fractures involves surgery along with metallic nails to hold pieces of bone together [15], [16], [17], [18], [19]. However, even under these conditions a common problem is non-union or delayed union of the fracture site resulting in impaired healing. Recombinant BMP2 and BMP7 proteins are used routinely for fracture healing treatment [20], [21]. BMPs have multiple effects on tissues other than bone and exert their effects through finely regulated events involving local production, storage and activation at appropriate time and site. Hence systemic administration of BMPs may cause unwarranted side effects. Further, due to rapid clearance of BMPs by the circulatory system its systemic residence time in vivo is very short [22]. Therefore, for clinical purposes, specialized delivery devices are used for in situ BMP delivery [23], [24]. Also BMPs have very short half lives in vivo [25]. The labile nature of BMPs coupled with the necessity to use specialized devices for BMP delivery for clinical purposes makes this therapy very expensive and limited in its scope.

A small molecule agonist of BMP signaling might serve as a substitute for clinical applications of BMPs to circumvent the issues associated with the use of BMP protein for therapeutic purposes. Such molecules may be identified through a high-throughput functional genetic or chemical genetic screen using a BMP-responsive reporter cell line. Although different BMP-responsive reporter cell lines have been previously reported in the literature these cell lines suffer from certain shortcomings such as lack of internal control and prolonged exposure time required for response to exogenous BMP [11], [12], [13], [14]. These limitations may potentially increase the number of false positives identified in a screen. Thus we have established a BMP responsive reporter cell line to address some of these shortcomings. This cell line is constructed by stably transfecting a BMP responsive dual luciferase reporter construct in the immortalized calvarial osteoblast cells isolated from tamoxifen inducible *Bmp2*; *Bmp4* double conditional knockout mouse strain. In this cell line the endogenous level of BMP signaling can be reduced by treatment with 4-hydroxytamoxifen (4-OHT) to increase the sensitivity of the assay. The reporter construct contains BRE driven Firefly Luciferase gene (FFLuc) and SV40 promoter/enhancer driven Renilla luciferase (RRLuc) gene, to serve as an internal normalization control for cell number as well as non specific transcription activation. This cell line, named BRITER (BMP Responsive Immortalized Reporter cell line), undergoes osteogenic differentiation in response to exogenous BMP and specifically reports BMP signaling activity in a robust and sensitive manner.

## Materials and Methods

### Generation of tamoxifen inducible *Bmp2*; *Bmp4* double conditional knockout mice

*Bmp2*; *Bmp4* double conditional knockout mouse strain (*Bmp2^C/C^*; *Bmp4^C/C^*) has been described before [6]. Tamoxifen inducible B6;129-*Gt(ROSA)26Sor^tm1(cre/ERT)Nat^*/J mouse strain, was imported from Jackson Laboratories, U.S.A. (Stock no. 004847) [26]. Here after this strain is referred to as R26CreER. Tamoxifen inducible *Bmp2*; *Bmp4* double conditional knockout mouse strain was generated in this study. *Bmp2^C/C^*; *Bmp4^C/C^* was crossed with R26CreER for two rounds to generate *Bmp2^C/C^*; *Bmp4^C/C^*; R26creER/R26CreER. This strain behaved as wild type and can be rendered BMP2 and BMP4 deficient upon administration of Tamoxifen (2.5 mg/20 g body weight for seven days) (data not shown).

All animal experiments were done according to the protocol approved by institute animal ethics committee which is registered with CPCSEA (reg. no. 810/03/ac/CPCSEA, dated 15/10/2003).

### Construction of BMP responsive reporter vector

BMP Responsive Element sequence was described and characterized by Korchynski et.al [10]. This BRE sequence was cloned in pGL3-Promoter vector (Promega). Briefly, two oligos as described below were synthesized.

BRE For NheI 5′-3′

CTAGCTCAGACCGTTAGACGCCAGGACGGGCTGTCAGGCTGGCGCCGGATCTAGCTCAGACCGTTAGACGCCAGGACGGGCTGTCAGGCTGGCGCCGA

With *NheI* overhang and BRE Rev BglII 5′-3′

GATCTCGGCGCCAGCCTGACAGCCCGTCCTGGCGTCTAACGGTCTGAGCTAGATCCGGCGCCAGCCTGACAGCCCGTCCTGGCGTCTAACGGTCTGAG

with *BglII* overhang.

The oligos were annealed and cloned in pGL3-Promoter vector between *NheI* and *BglII* sites to create pGL3-BRE-FFLuc vector. BRE-FFLuc cassette was excised from pGL3-BRE-FFLuc using *NheI* and *BamHI* restriction enzymes and cloned into pGL4.28 vector (Promega) between *NheI* and *BamHI* restriction sites to create pGL4.28-BRE-FFLuc vector.

Internal Ribosomal Entry Site (IRES) was PCR amplified from pCAGIG vector (Addgene No. 11159) using Pfu DNA polymerase with primers

AB383-Forward (5′-AATAGCC**GCTACGTAAATTCCG**-3′) and AB384-reverse (5′-TACGCGTTAGCCGTCATATG **ATATTATCATCGTGTTTT**-3′) where nucleotides denoted in bold letters indicate IRES specific sequence and reverse primer contains *MluI* (single underlined) and *NdeI* (double underlined) restriction enzyme sites. The blunt ended PCR product was cloned into *PmeI* linearized pGL4.28-BRE-FFLuc and the construct was named pGL4.28-BRE-FFLuc-IRES. Subsequently Renilla luciferase coding region was PCR amplified from pGL4.84 vector (Promega, U.S.A.) using primers

**Figure 1:**
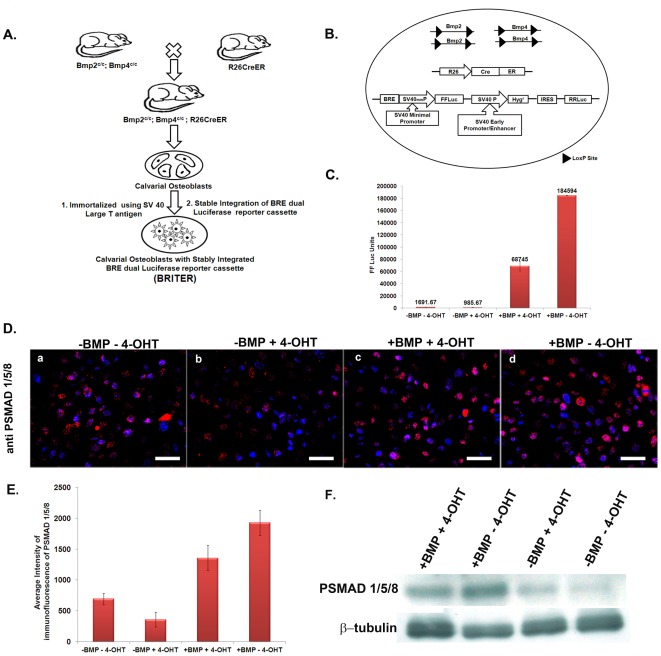
Creation of BRITER Cell Line. (**A**) Schematic showing steps involved in the creation of BRITER Cell Line. (**B**) Schematic showing critical genetic components of BRITER Cell Line. (**C**) BMP2 dependent FFLuc activity of BRITER Cell Line under four different conditions, namely “−BMP. −4-OHT”, “−BMP, +4-OHT”, “+BMP, +4-OHT” and “+BMP, −4-OHT”. (**D**) Anti-PSMAD 1/5/8 immunofluorescence in BRITER cell line under four different conditions: (**a**) −BMP−4-OHT, (**b**) −BMP+4-OHT, (**c**) +BMP+4-OHT and (**d**) +BMP−4-OHT. Scale bar 100 µm. (**E**) Quantification of Anti-PSMAD 1/5/8 immunofluorescence by Image J. (**F**) Western blot analysis of BRITER cell extracts cultured under indicated conditions with PSMAD 1/5/8 antibody. β-tubulin antibody has been used as loading control. Data shown are means ± SEM of three independent experiments carried out in triplicates.

AB381-Forward (ATAATAGCATATG **GCTTCCAAGGTGTACGAC** ) containing *NdeI* restriction enzyme site (single underlined) and AB382-reverse (ATAAATTACGCGT **TTAGACGTTGATCCTGGCG** ) containing *MluI* restriction enzyme site (single underlined). Purified PCR product was digested and cloned into *NdeI* and *MluI* site of pGL4.28-BRE-FFLuc-IRES construct resulting in the creation of BMP responsive dual luciferase reporter construct pGL4.28-BRE-FFLuc-IRES-RRLuc or pBFIR (Figure 1).

### Isolation of calvarial osteoblast cells

Primary calvarial osteoblasts were isolated as previously described [20]. Briefly, calvaria were isolated from 2–3 day old neonatal mice, washed with phosphate buffered saline (PBS) followed by digestion with 0.1% collagenase and 0.2% dispase at 37°C (five times for 10 minutes each). The last three groups of fractionated cells were collected and maintained in α-Minimum Essential Medium (Sigma Aldrich) containing 10% fetal bovine serum (FBS) (Invitrogen, U.S.A.) with 100 U/ml penicillin G and 100 µg/ml streptomycin and cultured at 37°C in a humidified atmosphere of air containing 5% CO_2_. Unless otherwise mentioned, α-MEM with 10% FBS, 100 U/ml penicillin G and 100 µg/ml streptomycin is referred to as the standard media in this report.

### Retroviral Immortalization of primary osteoblast cells

Primary calvarial osteoblast cells were immortalized through infection with a retrovirus expressing SV40 large T-Antigen [27]. Briefly, 293T cells were co-transfected with pBabe-Puro-SV40 LT retroviral vector [28] and pCL-10A1 packaging vector (Imagenex, U.S.A.) using Superfect transfection reagent following manufacturer's protocol (Fermentas). Fresh media was added 24 hours after transfection and the culture supernatant containing retrovirus was collected every 24 hours, filtered and was directly used to infect the primary osteoblast cells at passage three. 48 hours after infection, virus containing media was replaced with fresh media. 72 hours post-infection, osteoblast cells were cultured in standard medium supplemented with 2 µg/ml of puromycin (Sigma Aldrich) for another two weeks. Well isolated drug resistant colonies were further re-plated at very low density to obtain single cell derived colonies. Several colonies were expanded into cell lines and eventually the one displaying osteogenic activity comparable to uninfected primary osteoblasts was chosen for further manipulation. This Tamoxifen inducible *Bmp2*; *Bmp4* double conditional knockout osteoblast cell line is named BDO17 (Bmp2/4 Depletable Osteoblast Clone #17) cell line.

### Creation of BMP responsive reporter osteoblast cell line

BMP responsive dual luciferase reporter vector, pBFIR, was linearized with *BamHI* and transfected into BDO17 cells. Briefly, 20 µg of linearized pBFIR was transfected into the BDO17cells in a 100 mm plate using standard calcium phosphate method [29]. 24 hours later, cells were replenished with fresh media and allowed to grow for 48 hours. Transfected osteoblast cells were cultured in standard medium supplemented with 100 µg/ml of Hygromycin B (Invitrogen, U.S.A.) for another two weeks. Well isolated drug resistant colonies were further re-plated at very low density to obtain single cell derived colonies. Several clones were expanded into cell lines and checked for their BMP responsiveness after several passages and freeze thaw cycles. Based on maximal responsiveness of FFLuc and minimal responsiveness of RRLuc to exogenously added BMP, clone number 7 was used for further study. Hereafter, this cell line is referred to as BRITER (BMP Responsive Immortalized Reporter cell line). Most of the assays with BRITER cells were conducted at 25 passages unless otherwise mentioned.

### Alkaline phosphatase activity

For detecting alkaline phosphatase activity [30], [31], BRITER cells were cultured in 12-well plate containing mineralization medium (Standard medium containing 50 µg/ml ascorbic acid and 10 mM sodium β-glycerophosphate) for 2 weeks. Where indicated, cells were treated with 4-OHT (1 µM) and/or BMP2 (100 ng/ml) (Sino Biologicals, China) for the duration of the experiment. After two weeks of culturing, cells were washed with PBS. 500 µl of Fixation solution (citrate buffer, 60% acetone, 10% methanol) was added to each well and kept at room temperature for 5 minutes. The fixed monolayer was washed with cold distilled water and incubated with 5-Bromo-4-chloro-3-indolyl phosphate/Nitroblue tetrazolium chloride (BCIP/NBT) liquid substrate system (Roche) [1 µl of NBT (50 mg/ml)+1 µl of BCIP (25 mg/ml) in 1000 µl of NTM buffer, pH 9.5]. The reaction was stopped by removing the substrate solution and washing 3 times with cold distilled water. Cells were examined under the microscope (Leica DMIL-LED) and photographed (Leica DFC290).

For quantitative estimation of alkaline phosphatase activity we performed colorimetric assay using a chromogenic substrate of alkaline phosphatase, namely p-nitrophenyl phosphate (pNPP, Sigma Aldrich, Cat No. P7998). The assay was conducted as per manufacturer's protocol. In short, 10^6^ BRITER cells were plated in 6 cm dishes containing mineralization medium (Standard medium containing 50 µg/ml ascorbic acid and 10 mM sodium β-glycerophosphate) and cultured for 2 weeks. Where indicated, cells were treated with 4-OHT (1 µM) and/or BMP2 (100 ng/ml) (Sino Biologicals, China) for the duration of the experiment. After two weeks of culturing, cells were washed with PBS, 1 ml of pNPP, ready-to-use, substrate was added to each of the plates. The plates were incubated at 37°C for 30 minutes. The production of p-nitrophenol was measured at 405 nm absorbance in microplate reader. Alkaline phosphatase activity is expressed as nmol of product/minute/10^6^ cells. 1 absorbance unit is equal to 64 nmol of p-nitrophenol. The assay was done in triplicate for all the four different culture conditions.

### Alizarin red and von Kossa staining

For alizarin red staining, BRITER cells were fixed in cold 70% ethanol for 1 hour at 4°C. The fixed monolayer was washed with cold distilled water and stained with a fresh 2% Alizarin Red S solution (pH 4.2) for 5 minutes at room temperature [32]. The cell layers were then washed with deionized water and photographed. For von Kossa staining [33], the cells were rinsed with PBS and fixed with 10% neutral buffered formalin solution for 30 minutes. After washing with water, the cells were incubated with freshly prepared 5% silver nitrate solution in distilled water for 45 min at room temperature under 100 watt light source. The silver nitrate stain was developed with freshly prepared 5% sodium carbonate in 25% neutral buffered formalin for 2–5 minutes, until the bone nodules turned black. Finally, the cells were washed again with water and treated with 5% sodium thiosulphate for 2 minutes. The samples were rinsed in water and the bone nodules were observed under the microscope and photographed.

### RNA preparation and reverse transcription-polymerase chain reaction

BRITER cells were cultured in mineralization media for 2 weeks. Total RNA was extracted using Tri-reagent (Sigma Aldrich) and treated with DNAseI (Promega U.S.A.). 5 µg of total RNA was reverse transcribed into cDNA using ImProm-II™ Reverse Transcription System (Promega U.S.A.). Specific primers for the RT-PCR of osteoblast markers e.g. Osterix (Osx), collagen, type I, alpha 1 (Col1A1), Bone sialoprotein (BSP) Runt-related transcription factor 2 (Runx2), Osteocalcin (OCN), Alkaline Phosphatase (ALP) and Gapdh are described in (Table S1). A primer pair for SV40 large T-Antigen was also used to determine stable integration and expression of the immortalizing gene.

### 4-Hydroxytamoxifen mediated recombination

BRITER cells were cultured in 100 mm tissue culture plates with and without 4-Hydroxytamoxifen (1 µM) for 24 hours. Genomic DNA and total RNA was isolated from the cells. Genomic DNA PCR was carried out using primers described in Table S1 and Figure S2A. Change in the abundance of *Bmp2* and *Bmp4* mRNA was examined through RT-PCR using primers described in Table S1. Amplicon for Cre transgene was used a loading control for genomic DNA PCR while Gapdh mRNA was used as a loading control for RT-PCR experiment.

### Phospho-SMAD 1/5/8 immunofluorescence

BRITER cells were plated in 12 well tissue culture plate and treated with 4-OHT (1 µM) and/or BMP2 (100 ng/ml) for 24 hours as indicated. Cells were washed with PBS followed by treatment with hydrogen peroxide (3%) for 5 minutes at room temperature. After washing with PBT (PBS+ 0.5% Tween-20) cells were incubated in blocking solution (5% heat inactivated goat serum in PBT) for 1 hour at room temperature. Primary antibody against PSMAD 1/5/8 (Cell Signaling, U.S.A.) was added at 1∶100 dilution in blocking solution for 16 hours at 4°C. Cells were washed with PBS and incubated with Cy3-conjugated anti-rabbit IgG (Jackson Immunoresearch, U.S.A.) in PBS for 2 hours at room temperature. After washing with PBS, cells were counterstained with DAPI and mounted in Vectashield antifade reagent (Vector Laboratories, U.S.A.). Quantification of average PSMAD 1/5/8 immunoreactivity was carried out using Image J software (NIH). For each analysis three independent plates were included for each of the four culture conditions and five different fields were photographed from each of the plates. The experiment was repeated three times. The data is presented as average intensity of anti-PSMAD 1/5/8 immunofluorescence.

### Phospho SMAD 1/5/8 Western blot

For Western blot detection of PSMAD 1/5/8, lysates were prepared from BRITER cells cultured as above. The lysis buffer (50 mM Tris, pH 7.5, 150 mM NaCl, 1% NP-40, 1% DOC, 0.1% SDS 1 mM PMSF and 1 mM EDTA) contained protease inhibitor cocktail (Sigam-Aldrich) and phosphatase inhibitor cocktail (Calbiochem). Total protein from each lysates was estimated using Bradford method. 30 µg protein for each sample was resolved by SDS-PAGE (10%). The resolved proteins were transferred onto nitrocellulose membrane and blocked with 5% BSA. PSMAD 1/5/8 was detected by incubating the membrane with antibody against PSMAD 1/5/8 (Cell Signaling, U.S.A.) at 1∶1000 dilution for 4 hours at room temperature followed by incubation with 1∶2000 dilution of anti-rabbit immunoglobulin coupled to Horse Radish Peroxidase (Jackson Immunoresearch). The signal was detected by enhanced chemiluminescence detection kit (ECL, Pierce). Anti-β-tubulin (1∶5000, Sigma) was used as loading control.

### Dual Luciferase Assay

For C2C12 cells, the cells were transiently transfected with pBFIR dual luciferase reporter construct using Fugene-HD (Promega) following manufacturer's protocol. The transfected cells were cultured in standard medium for 24 hours. After 24 hours the culture medium was changed to α-MEM containing 0.1% FBS. Six hours later, cells were supplemented with 100 ng/ml of recombinant BMP2 protein for indicated time periods. Dual luciferase assay was carried out as per manufacturer's recommendation (Promega U.S.A). Three independent experiments were done in triplicates. It should be noted that the cells were maintained in α-MEM containing 0.1% FBS during the incubation period with recombinant BMP2 protein.

BRITER cells were plated in 24 well culture plate supplemented with standard medium. When the cells were 70% confluent, the wells were supplemented with regular medium containing 1 uM 4-OHT or vehicle (Ethanol). 18 hours later the culture medium was changed to α-MEM containing 0.1% FBS and 4-OHT as indicated. Six hours later cells were supplemented with indicated amount of recombinant BMP2 protein for indicated length of time. Dual luciferase assay was carried out as per manufacturer's recommendation (Promega U.S.A). All experiments were done in triplicates. It should be noted that cells were maintained in α-MEM containing 0.1% FBS and 4-OHT (where indicated) during the incubation period with recombinant BMP2 protein.

## Results

### Design and construction of BRITER cell line

The objective of this work was to construct a reporter cell line that will specifically report BMP signaling activity for the purpose of high-throughput functional genetic or chemical genetic screening of BMP signaling modifiers. For this purpose we wanted to take advantage of a well characterized BMP responsive enhancer named BRE [10]. The response of osteoblast cells to BMP signaling is very well studied [34], [35]. Recently, a transgenic mouse expressing BRE-lacZ was reported [36]. This mouse was shown to express beta-galactosidase gene in the developing mouse bone demonstrating that BRE is active in osteoblast cells in vivo. We therefore, went ahead to use osteoblast cells for the purpose of constructing the reporter cell line. However, osteoblast cells themselves are known to produce a variety of BMPs [37], [38] and production of endogenous BMP by the reporter cell line is expected to reduce its sensitivity towards exogenously added BMP as well as reduce the dynamic range of the assay. Therefore, we decided to engineer an osteoblast cell line which can be depleted of endogenous BMPs to a large extent. For this purpose, we generated an immortalized calvarial osteoblast cell line from tamoxifen-inducible *Bmp2*; *Bmp4* double knockout mouse strain. This cell line was named as BDO17 (Please see Materials and Methods and Figure 1A).

FFLuc activity provides quantitative readout and is amenable to high throughput assays. Further, it has been used earlier as readout of BRE activity in osteogenic cells [11]. We therefore cloned FFLuc gene downstream of BRE enhancer. In published reports, BRE responsiveness to exogenously added BMP has been assessed by normalized FFLuc activity i.e. the ratio of FFLuc activity in the presence and absence of exogenously added BMP [11], [12], [13], [14] (also see Table 1). This is a very sensitive and reliable assay for BMP activity if pure BMP is used. However, in a chemical or molecular genetic screen, cellular components other than BMP signaling pathway may be perturbed, e.g. basic transcription, translation, cell proliferation etc. This would give rise to altered FFLuc activity in treated cells compared to untreated cells leading to false-positive indications. To circumvent this problem it is essential to have an inbuilt internal control. To the best of our knowledge no BMP-responsive osteogenic cell line has been generated till date which has such an in-built internal control. The most commonly used internal control for such purposes is to use Renilla luciferase (RRLuc) gene driven by a constitutive promoter. In the published literature dual luciferase assays are generally conducted by co-transfection of two constructs, one containing FFLuc gene and the other containing RRLuc gene. However, in view of the poor transfection efficiency of BDO17 cells and to ensure the reproducibility of the assay we decided to combine both the reporter genes in the same construct. However, in such a construct the RRLuc gene would be in close proximity to BRE enhancer and therefore a possibility exists that RRLuc activity would also become responsive to exogenously added BMP protein. To examine this possibility, the dual luciferase construct was first transiently transfected in C2C12 cells and tested for two criteria: (1) BRE-FFLuc is responsive to exogenously added BMP2 and (2) RRLuc activity driven by SV40 promoter is not responsive to exogenously added BMP. We observed that the stimulation of relative luciferase activity in response to exogenously added BMP is approximately 18 fold comparable to an earlier described report where dual luciferase assay was conducted by transient co-transfcetion of two constructs [36] in C2C12 cell line (Blue line, Figure S1). We observed 4 fold stimulation of normalized luciferase activity upon addition of exogenous BMP2 protein to C2C12 cells transiently transfected with the dual luciferase construct (Red line, Figure S1). The normalized luciferase activity observed with our dual luciferase construct is comparable to some of the earlier reported cell lines where the assay was conducted in C2C12 cells stably transfected with BRE-FFLuc construct [12], [14] (also see Table 1). Further, we did not observe any significant change in RRLuc activity upon BMP addition (Green line, Figure S1). Once the construct was verified, it was stably integrated into BDO17 cells to generate BRITER cell line (BMP Responsive Immortalized Reporter cell line).

**Table 1:** Comparison of BRITER with previously reported osteogenic BMP reporter cell Lines.

S No.	Cell Line	BMP (ng/ml)	Fold Induction (Normalized FF Luc)	Fold Induction (Relative Luc)	Time (Hrs.)	Internal Control	Normalized by	Ref.
1	C2C12 Cells stably transfected with BRE-FFLuc construct	50	5	N/A	16	None	Untreated FFLuc value	12
		10	2	N/A	16	None		
2	C2C12 Cells stably transfected with BRE-FFLuc construct	19.5	1	N/A	15	None	Untreated FFLuc value	14
								
3	C2C12 Cells stably transfected with BRE-FFLuc construct	100	35	N/A	24	None	Untreated FFLuc value	13
		0.5	2	N/A	24	None		
4	BRITER( BMP depletable osteoblast, stably transfetcted with dual Luciferase construct)	100	35-50	45-100	3	SV-40 RR-Luc	SV40-RRLuc as well as untreated FFLuc values	In this study
		10	N/A	17	3	SV-40 RR-Luc		

### Characterization of BRITER cell line

**Figure 2:**
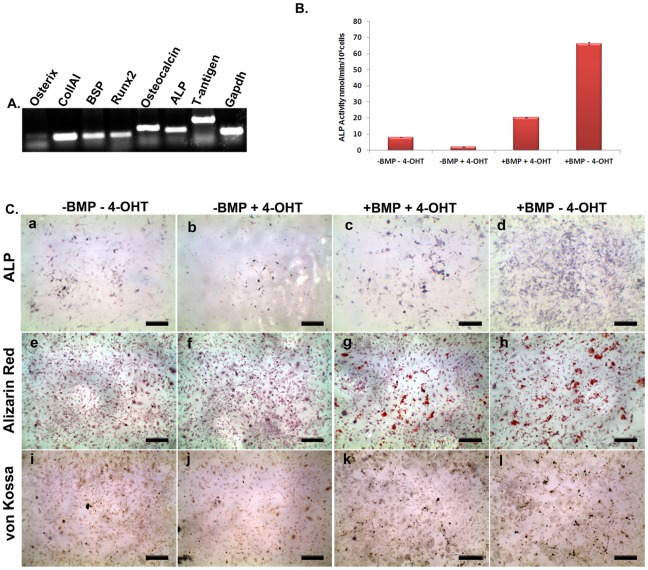
Characterization of BRITER cell line. (**A**) RT-PCR analysis of BRITER cell line for osteblast specific marker genes: Osterix (lane 1), ColIAI (lane 2), BSP (lane 3), Runx2 (lane 4), Osteocalcin (lane 5), Alkaline Phosphatase (lane 6), Large T antigen (lane 7) and GAPDH (lane 8), respectively. (**B**) Quantification of ALP activity of BRITER cell extracts cultured under indicated conditions. (**C**) Alkaline Phosphatase staining (**a–d**). Alizarin red staining (**e–h**). von Kossa staining (**i–l**) of BRITER cell line cultured under following conditions “−BMP−4 OHT” (**a, e, i**), “−BMP+4-OHT” (**b, f, j**), “+BMP+4 OHT” (**c, g, k**) and “+BMP−4-OHT” (**d, h, l**). Scale bar 10 mm. Data shown is means ± SEM of experiments carried out in triplicates.

We first wanted to investigate whether BRITER cells can be depleted of endogenous BMP activity upon 4-OHT treatment. For this purpose we cultured the cells for 24 hours in presence or absence of 4-OHT. Genomic DNA and total RNA was isolated from the 4-OHT treated and untreated cells to investigate recombination of *Bmp2* and *Bmp4* loci as well as abundance of *Bmp2* and *Bmp4* mRNA. We observed that 24 hours of 4-OHT treatment caused significant recombination of *Bmp2* and *Bmp4* loci (Figure S2B) along with concomitant decrease in the corresponding mRNA molecules (Figure S2C). To assess the status of BMP signaling in these cells, before and after BMP depletion, we cultured the cells for 24 hours under the following four conditions: (1) in the absence of exogenous BMP2 protein as well as 4-OHT (−BMP2−4-OHT), (2) in the absence of exogenous BMP2 protein but with 4-OHT (−BMP2+4-OHT), (3) in the presence of exogenous BMP2 protein and 4-OHT (+BMP2+4-OHT) and (4) in the presence of exogenous BMP2 protein but absence of 4-OHT (+BMP2−4-OHT). In the conditions 3 and 4, the amount of BMP2 protein added was 100 ng/ml for a period of 3 hours. In these cells, cultured under the four conditions, we measured FFLuc activity as well as anti-PSMAD 1/5/8 immunoreactivity, a read out of active BMP signaling. We observed a basal level of FFLuc activity in the untreated cells (−BMP−4-OHT) which was reduced upon 4-OHT treatment (compare the bars for “−BMP−4-OHT” and “−BMP+4-OHT” in Figure 1C). It is likely that this reduction in FFLuc activity is due to the lowering of endogenous BMP levels by the addition of 4-OHT. We observed significant increase in FFLuc activity when BMP was added to 4-OHT treated cells (compare the bars for “−BMP+4-OHT” and “+BMP+4-OHT” in Figure 1C). Maximum FFLuc activity was observed when BMP was added to 4-OHT untreated cells (compare the bars for “+BMP+4-OHT” and “+BMP−4-OHT” in Figure 1C). Next we examined PSMAD 1/5/8 immunofluorescence (Figure 1D) in the cells cultured under different conditions and quantified the same using Image J software (Figure 1E). We observed a basal level of PSMAD 1/5/8 immunoreactivity in the untreated BRITER cells which was reduced upon 4-OHT treatment (compare Figure 1D, panel a with figure 1D, panel b and Figure 1E). We observed significant increase in PSMAD 1/5/8 immunoreactivity when BMP was added to 4-OHT treated cells. Maximum PSMAD 1/5/8 immunoreactivity was observed when BMP was added to cells not treated with 4-OHT (compare Figure 1D, panel c with Figure 1D, panel d and Figure 1E). We have also analysed the abundance of PSMAD 1/5/8 by Western blot analysis of lysates of BRITER cells cultured under the different conditions described above (Figure 1F). The Western blot data confirms that BRITER cells have low basal level of PSMAD 1/5/8 which may be stimulated by addition of exogenous BMP2 protein (compare lanes −BMP−4-OHT with +BMP−4-OHT). The maximum abundance of PSMAD 1/5/8 was observed when recombinant BMP2 protein was added to 4-OHT untreated BRITER cells (compare lanes +BMP−4-OHT with +BMP+4-OHT). We have observed a slight decrease in basal level FFLuc activity as well as PSMAD 1/5/8 immunofluorescene upon addition of 4-OHT to BRITER cells (see above). However, we could not detect any reduction of PSMAD 1/5/8 abundance upon 4-OHT addition by Western blot ((compare lanes −BMP−4-OHT with −BMP+4-OHT). The reason for this apparent anomaly is not clear. In this context it may also be noted that we have also observed depletion of endogenous level of ALP activity of BRITER cells upon 4-OHT treatment (please see below). We next characterized the BRITER cells for several criteria specific for osteoblast cell lines. Reverse transcription of total mRNA isolated from BRITER cells cultured for two weeks in mineralization media followed by PCR with primers specific for osteoblast marker genes revealed that BRITER cells express several known osteoblast markers e.g. Osx, ColIA1, BSP, Runx2, Ocn and ALP (Figure 2A). RT-PCR for SV40- large T antigen confirmed the integration and expression of the immortalizing gene in BRITER cells (Figure 2A).

To assess the differentiation and mineralization ability of BRITER cells we investigated the production of Alkaline Phosphatase (ALP), presence of Alizarin red and von Kossa positive mineralized matrix in BRITER cells by culturing in mineralizing media for two weeks. We have also quantified the ALP activity in the cells cultured under the different conditions (Figure 2B) using colorimetric method. While some ALP positive cells were observed in untreated cells after the culture period, less number of ALP positive cells were detectable upon depletion of endogenous BMP by 4-OHT treatment (compare panels a and b of Figure 2C, also see Figure 2B). The number of ALP positive cells increased significantly when BMP-depleted (cultured in presence of 4-OHT) cells were cultured in presence of exogenously added BMP2 protein (compare panels b and c of Figure 2C, also see Figure 2B). Maximum number of ALP positive cells were observed when BMP2 protein was added to cells wherein the endogenous BMP was not depleted, i.e. when cells were cultured in presence of BMP2 but absence of 4-OHT (compare panels c and d of Figure 2C, also see Figure 2B). Unlike the presence of ALP activity or PSMAD 1/5/8 immunoreactivity, we could not detect any alizarin red or von Kossa positive mineralized nodules in BRITER cells cultured in absence of exogenously added BMP. However, significant number of mineralized nodules were observed in BMP depleted cells cultured in presence of exogenously added BMP (compare panels e and f with panel g of Figure 2C for alizarin red staining and compare panels i and j with panel k of Figure 2C for von Kossa staining) which increased even further when cells were not depleted of endogenous levels of BMP i.e. cultured in absence of 4-OHT (compare panels g and h of Figure 2C for alizarin red staining and compare panels k and l of Figure 2C for von Kossa staining). Our observations taken together strongly suggest that BRITER cells are BMP-depletable and display BMP dependent osteogenic activity.

### Sensitivity of BRITER cells to exogenously added BMP

**Figure 3:**
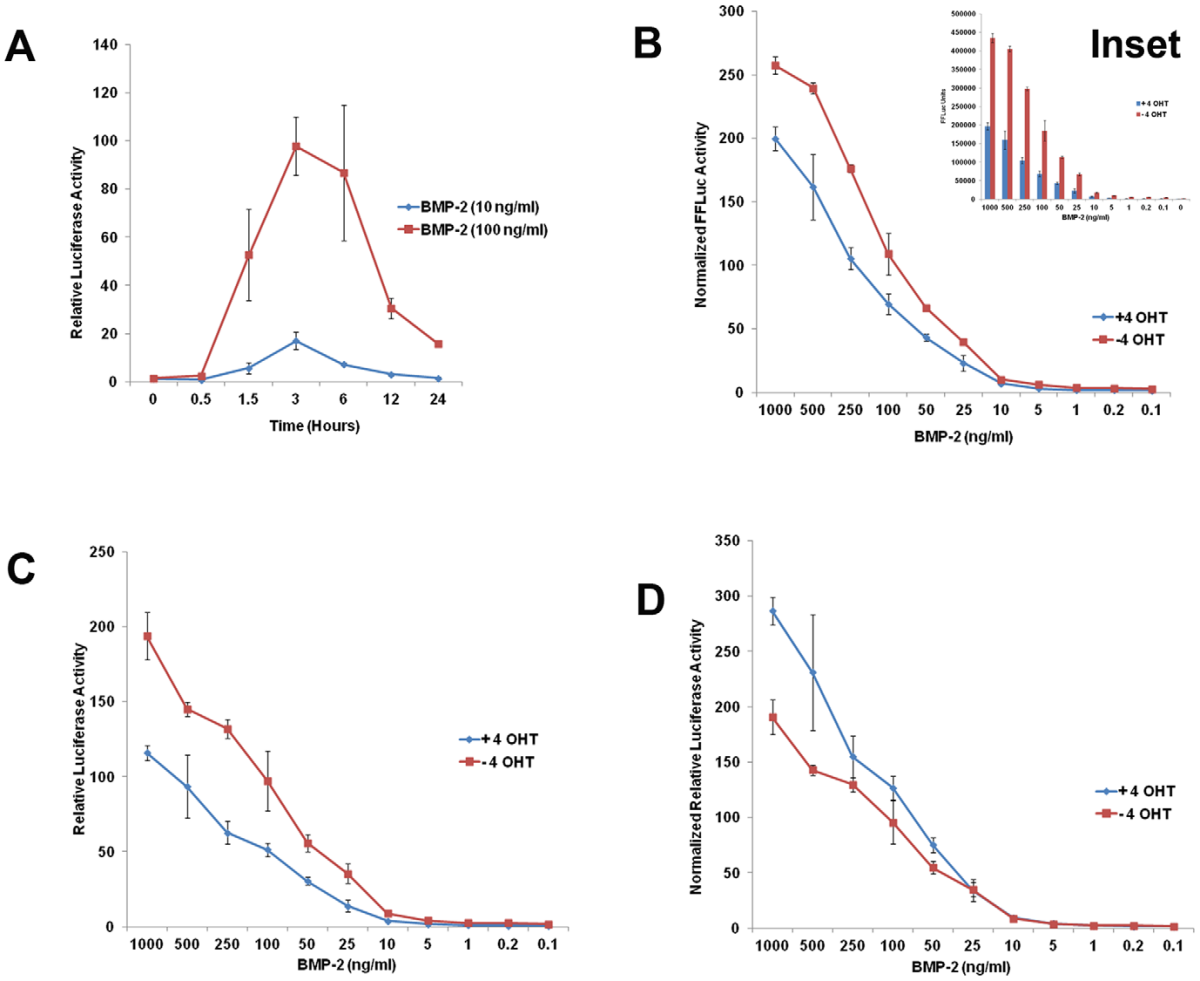
(**A**) Time course analysis showing relative FFLuc activity in presence of 100 ng/ml (red line) and 10 ng/ml (blue line) BMP2 concentrations at different time points indicated. (**B**) Normalized FFLuc activity, (Inset showing absolute values of FFLuc activity) after 3 hours of stimulation with different BMP2 concentrations in presence (blue line) or absence (red line) of 4-OHT. (**C**) Relative luciferase activity after 3 hours of stimulation with different BMP2 concentrations in presence (blue line) or absence (red line) of 4-OHT. (**D**) Normalized relative luciferase activity after 3 hours of stimulation with different BMP2 concentrations in presence (blue line) or absence (red line) of 4-OHT. Data shown are means ± SEM of three independent experiments carried out in triplicates.

We next wanted to investigate the responsiveness of BRITER cells to exogenously added BMP of varying concentrations for which it was necessary to identify the best time point to carry out this analysis. Thus we cultured BRITER cells in serum deprived media for 6 hours following which we added 100 ng/ml (High BMP) and 10 ng/ml (Low BMP) of BMP2 protein in two parallel sets of wells. We cultured the cells further for 0.5, 1.5, 3, 6, 12 and 24 hours after BMP addition. At the end of the intended culture duration dual luciferase assay was performed with BRITER cell lysate. Cells cultured in presence of high (Red line) or low BMP (Blue line) concentration displayed maximum BMP responsiveness 3 hours post BMP addition (Figure 3A). The FFLuc activity of BRITER cells decreased sharply between six hours and twelve hours of BMP2 protein addition (Figure 3A). This sharp decline in FFLuc activity of BRITER cells after six hours of BMP2 protein addition raised the possibility that the BRE enhancer in BRITER cells gets sensitized upon prolonged exposure to BMP2 protein. Alternatively, it is also possible that under the assay conditions BMP2 activity decreases with time. To distinguish between these two possibilities we have conducted another time course experiment where FFLuc activity of BRITER cells were measured after 0, 1, 1.5, 3, 4.5, 6, 7.5 and 9 hours after 100 ng/ml BMP2 protein addition. In a parallel experiment, we added recombinant BMP2 protein (100 ng/ml) at 0 hour time point and then again at 6 hour time point and measured relative luciferase activity at 9 hour time point i.e. 3 hours after addition of the second dose of BMP2 protein (denoted as 9+ hour time point). The relative luciferase activity at 9+ hour time point is very similar to that at the 3 hour time point. If the BRE enhancer in BRITER cells became sensitized upon BMP2 protein exposure there would not have been any further stimulation of FFLuc activity upon BMP2 protein addition at the 6 hour time point. However, we observed significantly more FFLuc activity in the 9+ hour time point sample as compared to that of the 9 hour time point sample (Figure S3A). In fact the FFLuc activity of 9+ hour time point sample is close to that of the 3 hour time point sample (Figure S3A). This suggests that the sharp decline in FF-Luc activity in BRITER cells after 6 hours of BMP2 protein addition is a reflection of decline of BMP2 activity with time under the assay conditions and not a result of sensitization of BRE enhancer. It should also be noted that in this time course also the highest FFLuc activity was observed 3 hours after BMP2 protein addition (Figure S3A). Therefore we decided to assess BMP responsiveness of BRITER cells 3 hours post BMP2 protein addition in all subsequent experiments.

To ensure that BRITER has a stable phenotype we cultured BRITER cells for 25, 30, 35, 40 or 45 passages and froze several aliquots at all these different passages. We conducted dual luciferase assays with BRITER cells at these different passage numbers 3 hours post BMP2 protein addition. The relative luciferase activity produced by BRITER cells upon stimulation by BMP2 protein was highly comparable between all these different samples suggesting that BRITER cells have stable phenotype vis-à-vis response to exogenously added BMP2 protein (Figure S3B).

To investigate the sensitivity of BRITER cells to exogenously added BMP, we cultured BMP-depleted (24 hr. 4-OHT treated) or untreated BRITER cells for 3 hours in presence of exogenously added BMP2 protein with concentrations ranging from 0.1 ng/ml to 1000 ng/ml. Irrespective of whether the cells were BMP depleted or not, we observed significant stimulation of FFLuc activity only when the exogenously added BMP2 protein concentration was more than 5 ng/ml. The normalized FFLuc activity (FFLuc [+BMP]/FFLuc [−BMP]) increased linearly between 10 ng/ml to 1000 ng/ml of exogenously added BMP2 protein concentration. The normalized FFLuc activity was not saturated within the BMP dosage range of our investigation (Figure 3B). It should be noted that the normalized FFLuc activity in presence of any given concentration of BMP was always more when cells were not depleted of endogenous BMP. Further, the absolute value of FFLuc activity is significantly more if endogenous BMP activity is not depleted (*Inset*, Figure 3B).

Measuring normalized FFLuc activity in presence and absence of exogenously added BMP is a sensitive measure of exogenously added BMP activity. However, in the context of a drug screen or functional genetics screen, it is desirable to have an internal control to ensure that the variation in FFLuc activity is not an indirect consequence of altered cell proliferation or basal level of transcription due to chemical or genetic modification of the cell line. For this purpose, we plotted the relative luciferase activity (FFLuc/RRLuc) graph. This dosage response graph clearly demonstrates that RRLuc activity is not influenced by exogenously added BMP (Figure 3C). It should be noted that the relative luciferase activity in presence of any given concentration of BMP was always more when cells were not depleted of endogenous BMP.

While normalized FFLuc activity (FFLuc [+BMP]/FFLuc [−BMP]) is a measure of response to exogenously added BMP, it does not provide an estimation of non-specific activation of FFluc. On the other hand relative luciferase (FFLuc/RRLuc) activity reflects specific stimulation of BRE enhancer, it does not provide an estimation of endogenous (basal) BMP activity. To compensate for both these variables and to assess the sensitivity of BRITER cells with respect to exogenously added BMP, we plotted a graph of normalized relative luciferase activity using the formula (FFLuc [+BMP]/FFLuc [−BMP])/(RRLuc [+BMP]/RRLuc [−BMP]). It should be noted that, in contrast to Figure 3B and Figure 3C, normalized relative luciferase activity in presence of any given concentration of BMP was always more when cells were depleted of endogenous BMP. This graph clearly demonstrated that BRITER cells are relatively more sensitive to exogenously added BMP2 protein when endogenous BMP activity is depleted by 4-OHT mediated recombination of *Bmp2* and *Bmp4* loci (Figure 3D).

## Discussion

The objective of this work was to generate a robust, sensitive and internally controlled BMP reporter osteoblast cell line suitable for chemical or molecular genetic screen of BMP signaling modifiers. For this purpose we immortalized mouse calvarial osteoblast cells isolated from a tamoxifen inducible *Bmp2*; *Bmp4* double conditional knockout mouse strain and stably transfected it with a dual luciferase reporter construct. BMP reporter cell lines have been reported earlier in the literature [12], [13], [14] where BRE-FFLuc reporter construct has been stably integrated into C2C12 cell line. These cell lines provided very sensitive assays for bioactive BMP molecules. The sensitivity and robustness of our cell line is comparable to (if not more) the ones reported earlier. However, unlike the previously reported cell lines BRITER has an in-built internal control to enable specific detection of BMP activity modifiers. Also, the rapidity with which BRITER responds to exogenously added BMP protein suggests that stimulation of BRE-FFLuc in BRITER cells is a direct consequence of activation of BMP signaling. This is in stark contrast to the previously reported cell lines where BMP responsiveness was apparent only after 15–24 hours (Table 1). In the sections below we have discussed in detail, several aspects of the BRITER cell line such as robustness, sensitivity etc., which demonstrates that because of the genetic tools embedded in the cell line, BRITER is more appropriate for small molecule BMP agonist screening as well as functional genomic screening (comparison of BRITER cells with previously reported cell lines is presented in Table 1).

BRITER cells robustly respond to exogenously added BMP2 protein. We have observed a 94 fold and 51 fold stimulation of FFLuc activity in response to 100 ng/ml of exogenously added BMP2 protein in 4-OHT untreated and 4-OHT treated BRITER cells, respectively. To the best of our knowledge, the fold stimulation of reporter gene activity we observed with BRITER cell line is significantly more than the earlier reports [12], [13], [14]. Contrary to the previously reported cell lines, BRITER responds to exogenously added BMP2 protein within 1 hour with peak activity observed after 3 hours of BMP2 protein addition thus reducing the possibility of secondary factors influencing BRE-FFLuc activity [12], [13], [14].

In this context it is worth comparing the properties of C2C12 cells with that of an osteoblast derived cell line like BRITER. C2C12 cells are not intrinsically osteogenic i.e they cannot produce ALP activity unless stimulated by exogenously added BMP protein [39]. In contrast, an osteoblast derived cell line like BRITER can produce ALP activity on its own [40] (also see Figure 2C, panel a). This suggests that unstimulated C2C12 cells do not have all the components necessary to carry out an osteogenic program and gets reprogrammed to osteogenic fate upon exposure to exogenously added BMP while an osteoblast derived cell line like BRITER has all the components necessary to carry out an osteogenic program in response to BMP signal. This intrinsic difference between C2C12 cell line and BRITER cell line perhaps accounts for the stark difference in the kinetics of BMP responsiveness.

The readout of BMP signaling in BRITER cells is FFLuc activity. However, FFLuc activity may vary due to factors other than the ones affecting BMP signaling directly. BRE-FFLuc activity will depend on cell number and status of global gene expression. In the context of a chemical or molecular genetic screen, there may be factors affecting cell number and status of global gene expression thus giving false positive results. However in our construct, the production of Renilla luciferase is under the control of SV40 promoter, a non-BMP responsive constitutive promoter. Any change in the RRLuc activity would be indicative of a drug or genetic factor having activity not specific to BMP signaling pathway. We incorporated constitutive promoter driven RRLuc activity in BRITER to normalize for these non-specific factors. In our hands, under varying conditions RRLuc activity never varied more than 2 fold while FFLuc activity varied up to 300 fold strongly suggesting that BRITER cells can efficiently normalize for false positive indications and thus can significantly reduce downstream work following a screen. None of the previously reported osteogenic BMP-reporter cell lines contained such an in-built internal control (Table 1). Furthermore, BRITER responds to BMP in a dose dependent manner for upregulation of ALP activity (Figure 2B and Figure 2C). Thus ALP activity may serve as a secondary screen criteria further eliminating the need of extensive experimentation to narrow down the targets for further study.

BRITER cell line is designed for the purpose of chemical or molecular genetic screen of potential BMP signaling modifiers. It is therefore necessary to have a large dynamic range of the assay. Three lines of arguments taken together inspired us to create a cell line that can be depleted of endogenous BMP activity for the purpose of increasing the dynamic range of our assay system: 1. BMP signaling is necessary for bone formation [6], 2. BMP2 and BMP4 are two of the most critical BMPs for bone formation *in vivo* [6], and 3. Any osteogenic cell line is expected to produce endogenous BMP under osteogenic condition and hence may provide background BMP signaling activity. We used available *Bmp2*; *Bmp4* double conditional knockout mouse strains and tamoxifen inducible Cre expressing mouse strain to engineer the necessary genetic background for depleting endogenous BMP signaling to a large extent.

In this study we have defined sensitivity as a response to exogenous BMP indicated by the normalized relative luciferase activity (FFLuc [+BMP]/FFLuc [−BMP])/(RRLuc [+BMP]/RRLuc [−BMP]). Normalized relative luciferase value takes into account two variables that can affect the apparent sensitivity of BRITER cells to exogenously added BMP namely, endogenous BMP activity and non-specific stimulation of BRE-FFLuc activity. We observed that the sensitivity to exogenously added BMP is 1.5 to 2 fold higher (Figure 3D) upon depletion of endogenous BMP (+4-0HT). What was somewhat surprising is that the increased sensitivity is more apparent when exogenously added BMP concentration is 5 ng/ml or higher. When we conceived the experiment, we expected the sensitivity towards exogenously added BMP to be more at lower concentration of BMP (i.e. BMP depleted condition), however our observation is contrary to our expectations. This apparent contradiction may be a result of one of the two following factors

- The specific activity of BMP that we are using in these assays is not very high and the effect of exogenously added BMP is only apparent at a concentration higher than critical threshold of 5 ng/ml. However, this is unlikely to be the case as the fold change stimulation of FFLuc value with or without BMP addition is significantly more than the values reported in the literature earlier.
- Alternatively, it is possible that depletion of endogenous BMP for 24 hrs (by maintaining the cell line in presence of 4-Hydroxytamoxifen) causes depletion of other active molecules required for transcription and translation of Firefly luciferase upon BMP signaling. In fact, existing literature supports such a hypothesis. Runt-related gene 2 (*Runx2*), a downstream target gene of BMP signaling is shown to interact with SMAD1 and SMAD5 and regulate BMP signaling [41], [42], [43], [44]. Hence, lowering the levels of endogenous BMP can potentially lower the expression of Runx2 and/or other regulators of BMP signaling limiting the ability of BRITER cells to respond to BMP signaling below a critical threshold of exogenously added BMP concentration.

Cell based assays using luciferase reporter has been successfully used earlier for identification of novel drug candidates [45], [46], [47]. Similarly, cell based assays using luciferase reporter has been successfully used earlier for genome-wide RNAi screening to investigate several cellular pathways [48], [49], [50]. BRITER is thus equipped to be used for screening modifiers of BMP signaling pathway.

In conclusion, BRITER is highly sensitive to exogenously added BMP, responds robustly and specifically to active BMP signaling. This cell line is capable of undergoing osteogenic differentiation in graded response to active BMP signaling and is suitable for chemical and molecular genetic screens which may help elucidate the molecular players involved in BMP signaling pathway and/or identification of clinically important drug molecules.

## Supporting Information

Figure S1 **Time course of luciferase activity in C2C12 cells.** C2C12 cells were transiently transfected with dual luciferase construct (pBFIR) and treated with 100 ng/ml BMP2 protein or vehicle. Dual luciferase assay was conducted after indicated duration of incubation (in hrs). Green line depicts normalized (with BMP2 untreated cell lysate values) Renilla luciferase values. Red line depicts normalized (with BMP2 untreated cell lysate values) Firefly luciferase values. Blue line depicts relative luciferase (FFLuc/RRLuc) values.
(TIF)

Figure S2 **4-OHT induced recombination in BRITER cell line.** (**A**) Schematic showing the genomic configuration for *Bmp2* and *Bmp4* conditional alleles. The primers for genotyping are shown as red arrows. (**B**) *Bmp2* and *Bmp4* floxed alleles before and after recombination in absence and presence of 4-OHT, respectively. Amplicon for Cre transgene is used as loading control. (**C**) *Bmp2* and *Bmp4* mRNA levels before and after recombination in absence and presence of 4 OHT, respectively. RT-PCR of Gapdh mRNA has been used as loading control.
(TIF)

Figure S3 **(A) BRE enhancer does not become sensitized due to prolonged exposure to recombinant BMP2 protein.** Time course analysis showing relative FFLuc activity in presence of 100 ng/ml BMP2 concentrations at different time points indicated. For the “9+” hour time point sample recombinant BMP2 protein (100 ng/ml) was added at 0 hour time point and then again at 6 hour time point and relative luciferase activity was measured at 9 hour time point. (**B**) **BRITER has stable phenotype.** Relative luciferase activity of BRITER cells at different passage numbers (as indicated) was measured after treating the cells with 100 ng/ml of recombinant BMP2 protein for three hours. Relative luciferase activity of BRITER cells at passage number 25 (25 UT) and 45 (45 UT) were also measured with untreated cells.
(TIF)

Table S1 **Description of primers used in this study.**
(DOC)
