# Supplementary figures and images for: BRITER: A BMP Responsive Osteoblast Reporter Cell Line
Source: PLoS One. 2012 May 14;7(5):e37134. doi: 10.1371/journal.pone.0037134 (PMC3354957; doi:10.1371/journal.pone.0037134)

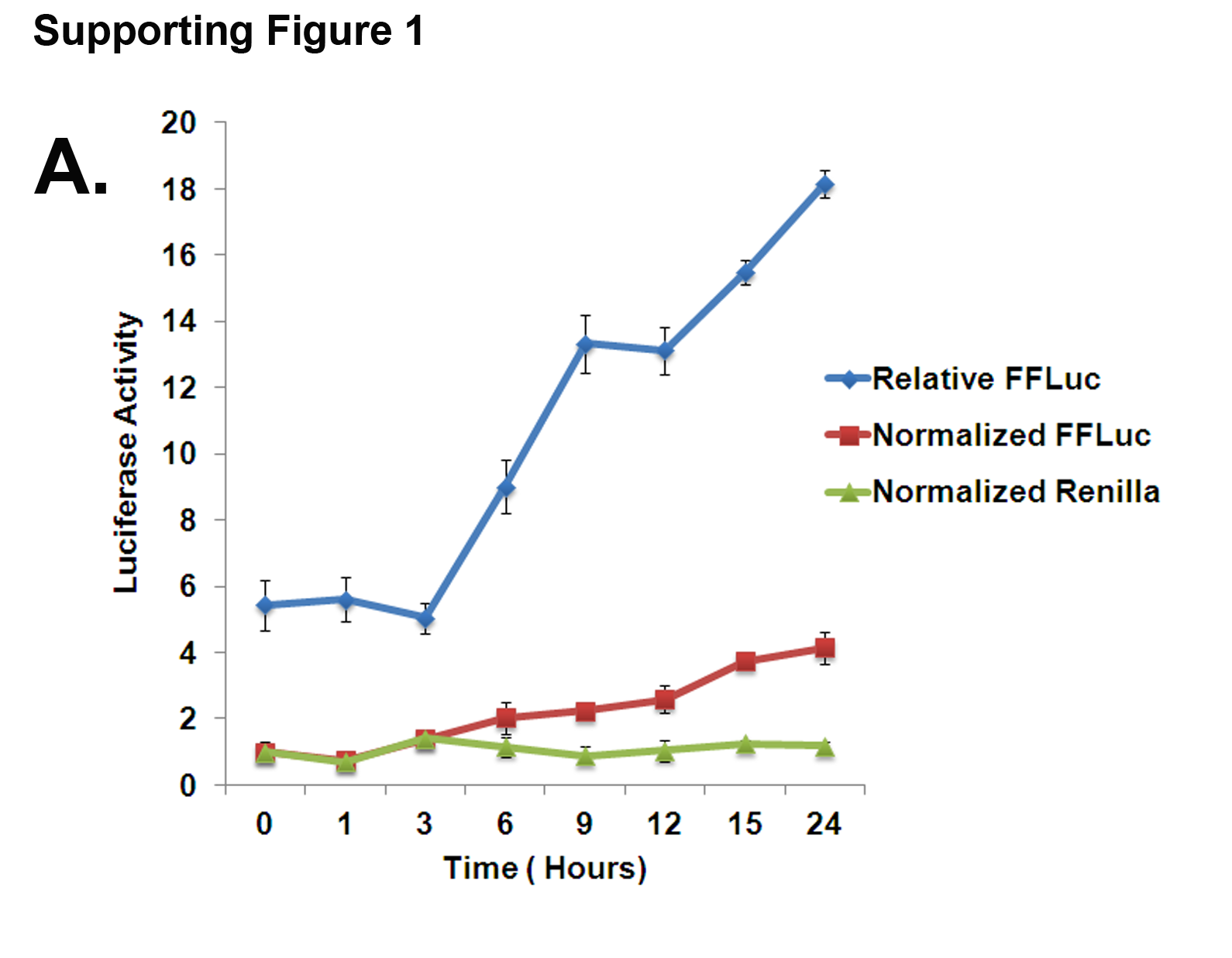

Supplement: Figure S1 — Time course of luciferase activity in C2C12 cells. C2C12 cells were transiently transfected with dual luciferase construct (pBFIR) and treated with 100 ng/ml BMP2 protein or vehicle. Dual luciferase assay was conducted after indicated duration of incubation (in hrs). Green line depicts normalized (with BMP2 untreated cell lysate values) Renilla luciferase values. Red line depicts normalized (with BMP2 untreated cell lysate values) Firefly luciferase values. Blue line depicts relative luciferase (FFLuc/RRLuc) values. (TIF) [file pone.0037134.s001.tif]

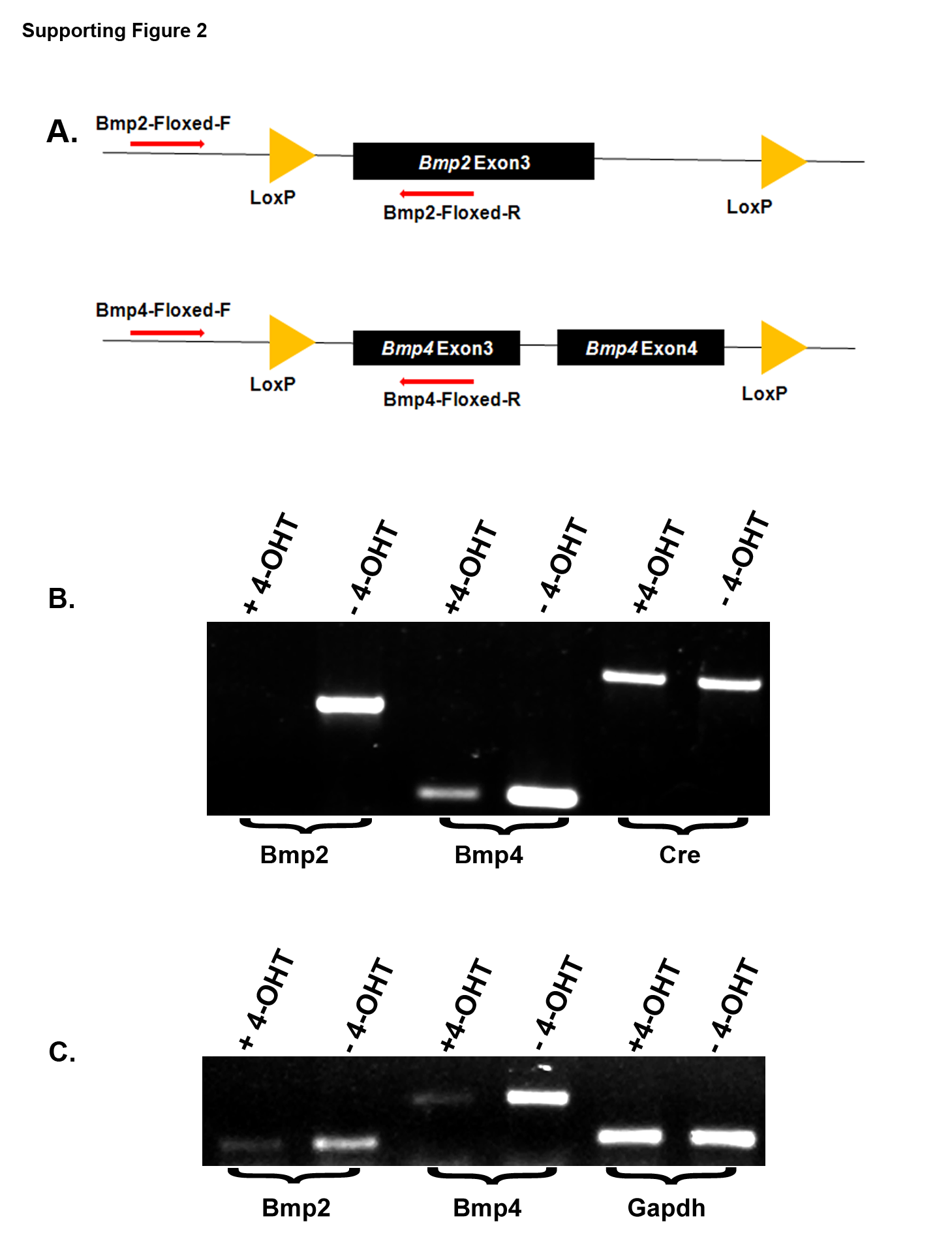

Supplement: Figure S2 — 4-OHT induced recombination in BRITER cell line. (A) Schematic showing the genomic configuration for Bmp2 and Bmp4 conditional alleles. The primers for genotyping are shown as red arrows. (B) Bmp2 and Bmp4 floxed alleles before and after recombination in absence and presence of 4-OHT, respectively. Amplicon for Cre transgene is used as loading control. (C) Bmp2 and Bmp4 mRNA levels before and after recombination in absence and presence of 4 OHT, respectively. RT-PCR of Gapdh mRNA has been used as loading control. (TIF) [file pone.0037134.s002.tif]

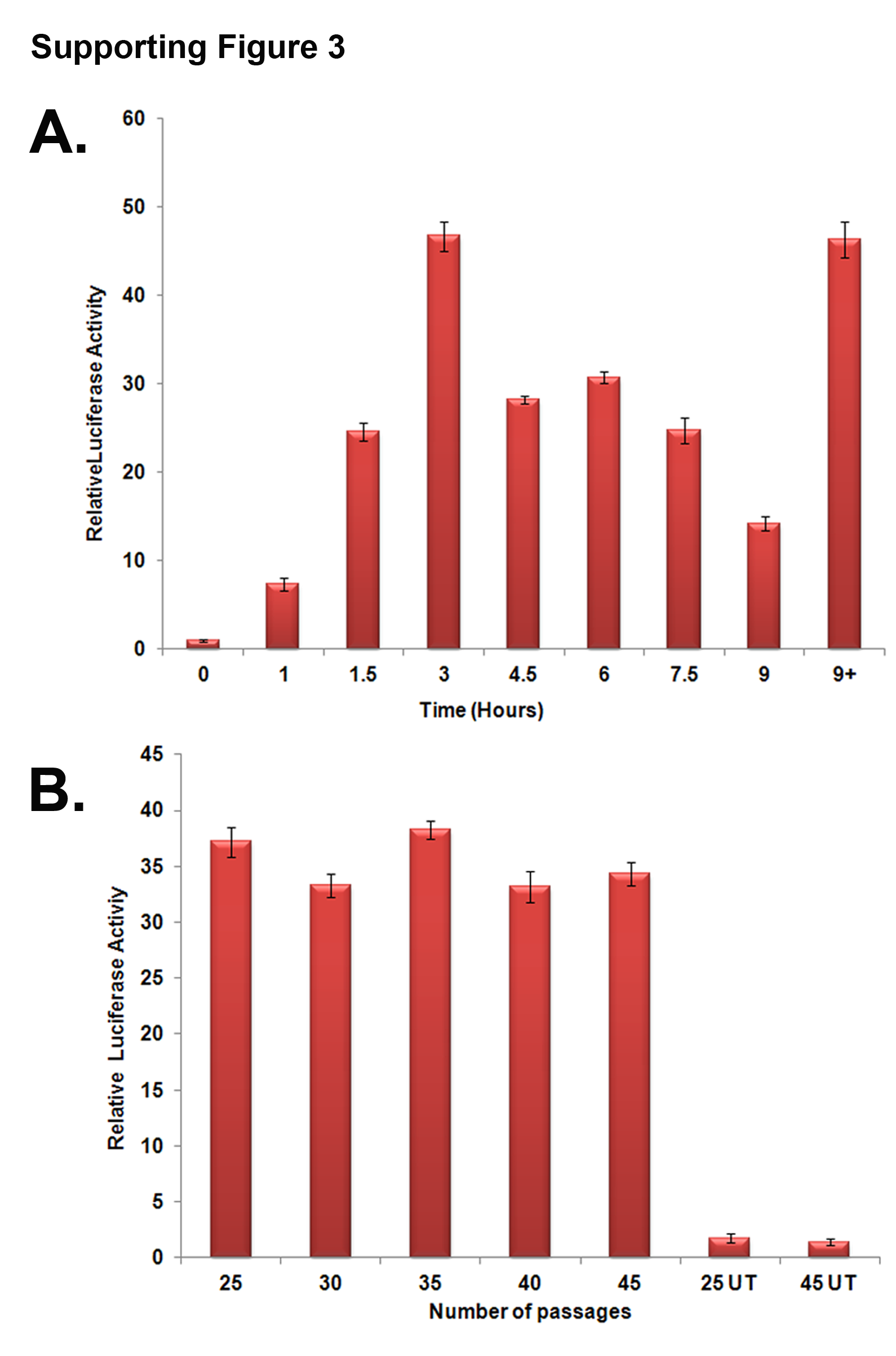

Supplement: Figure S3 — (A) BRE enhancer does not become sensitized due to prolonged exposure to recombinant BMP2 protein. Time course analysis showing relative FFLuc activity in presence of 100 ng/ml BMP2 concentrations at different time points indicated. For the “9+” hour time point sample recombinant BMP2 protein (100 ng/ml) was added at 0 hour time point and then again at 6 hour time point and relative luciferase activity was measured at 9 hour time point. (B) BRITER has stable phenotype. Relative luciferase activity of BRITER cells at different passage numbers (as indicated) was measured after treating the cells with 100 ng/ml of recombinant BMP2 protein for three hours. Relative luciferase activity of BRITER cells at passage number 25 (25 UT) and 45 (45 UT) were also measured with untreated cells. (TIF) [file pone.0037134.s003.tif]
