# Supplementary material for: BRITER: A BMP Responsive Osteoblast Reporter Cell Line
Source: PLoS One. 2012 May 14;7(5):e37134. doi: 10.1371/journal.pone.0037134 (PMC3354957; doi:10.1371/journal.pone.0037134)
Supplement: Table S1 — Description of primers used in this study. (DOC) [file pone.0037134.s004.doc]

**Table S**1

| **Primer name** | **Primer Sequence 5’🡪3’** |
| --- | --- |
| SV40 Large T-Antigen-F | AGCAGACACTCTATGCCTGTGTGGAGTAAG |
| SV40 Large T-Antigen-R | GACTTTGGAGGCTTCTGGATGCAACTGAG |
| Osterix-F | GAAGTCCAATGGGGATCTGA |
| Osterix-R | AGAATCCCTTTCCCTCTCCA |
| COLIA1-F | GAGCGGAGAGTACTGGATCG |
| COLIA1-R | GCTTCTTTTCCTTGGGGTTC |
| BSP-F | GAAGCAGGTGCAGAAGGAAC |
| BSP-R | ACTCAACGGTGCTGCTTTTT |
| Runx2-F | CCCAGCCACCTTTACCTACA |
| Runx2-R | TATGGAGTGCTGCTGGTCTG |
| ALP-F | CGGGACTGGTACTCGGATAA |
| ALP-R | TGAGATCCAGGCCATCTAGC |
| Osteocalcin-F | CTCTGTCTCTCTGACCTCACAG |
| Osteocalcin-R | CAGGTCCTAAATAGTGATACCG |
| BMP2-F | TGGAAGTGGCCCATTTAGAG |
| BMP2-R | TGACGCTTTTCTCGTTTGTG |
| BMP4-F | ACGTAGTCCCAAGCATCACC |
| BMP4-R | TCAGTTCAGTGGGGACACAA |
| GAPDH-F | ACCCAGAAGACTGTGGATGG |
| GAPDG-R | CACATTGGGGGTAGGAACAC |
| BMP2-Floxed-F | GTGTGGTCCACCGCATCAC |
| BMP2-Floxed-R | GGCAGACATTGTATCTCTAGG |
| BMP4-Floxed-F | AGACTCTTTAGTGAGCATTTTCAAC |
| BMP4-Floxed-R | AGCCCAATTTCCACAACTTC |
| CRE Recombinase-F | CGTACTGACGGTGGGAGAAT |
| CRE Recombinase-R | TGCATGATCTCCGGTATTGA |
